# Genome Sequence of *Methanobrevibacter* sp. Strain JH1, Isolated from Rumen of Korean Native Cattle

**DOI:** 10.1128/genomeA.00002-13

**Published:** 2013-02-28

**Authors:** Jong-Hwan Lee, Moon-Soo Rhee, Sanjay Kumar, Geun-Hye Lee, Dong-Ho Chang, Dae-Soo Kim, Sang-Haeng Choi, Dong-Woo Lee, Min-Ho Yoon, Byoung-Chan Kim

**Affiliations:** a Korean Collection for Type Cultures, Biological Resource Center, Korea Research Institute of Bioscience and Biotechnology (KRIBB), Yuseong-gu, Daejeon, Republic of Korea; b Genome Resource Center, KRIBB, Yuseong-gu, Daejeon, Republic of Korea; c Division of Applied Biology and Chemistry, Kyungpook National University, Daegu, Republic of Korea; d College of Agriculture & Life Sciences, Chungnam National University, Yuseong-gu, Daejeon, Republic of Korea

## Abstract

The *Methanobrevibacter* sp. strain JH1 was isolated from the rumen of Korean native cattle (HanWoo; *Bos taurus coreanae*). Here, we provide a 2.06-Mb draft genome sequence of strain JH1 that might provide more information about the lifestyle of rumen methanogens and about the genes and proteins that can be targeted to curb methane emissions.

## GENOME ANNOUNCEMENT

Methane, a potent greenhouse gas emitted from agriculture, represents ~40% of the emissions produced by anthropogenic activities. Among these, enteric fermentation has the maximum share in methane emissions. Mitigation strategies to reduce these emissions are not persistent (1). A diversity analysis of rumen methanogens revealed the dominance of the genus *Methanobrevibacter*, irrespective of locations, diets, breeds, etc. (2, 3). *Methanobrevibacter* sp. strain JH1 was isolated from the rumen of Korean native cattle, and this is the first example of pure isolation of a novel archaeal rumen methanogen from the Republic of Korea. The draft whole genome sequence of strain JH1 will reveal information about the major proteins and key genes that can be targeted for successful, long-term methane mitigation strategies with broad efficacy for the rumen.

The genome (252,070 reads totaling ~71.2 Mb, ~34-fold coverage of the genome) was analyzed using a whole-genome shotgun strategy with the Roche 454 Titanium sequencer for pyrosequencing. Quality filtered reads generated through Roche software were assembled *in silico* using the 454 Newbler 2.6 assembler, and 43 contigs >500 bp in size were obtained. These contigs were further assembled into 4 scaffolds (N_50_ scaffold size, 816 kb) based on the paired-end information. Gene prediction was performed using the Glimmer 3.02 modeling software (4), RNAmmer-1.2 (5), and the NCBI Clusters of Orthologous Groups (COG) database (6). Gene annotation and screening for noncoding ribosomal RNAs and transfer RNAs were carried out by the Rapid Annotations using Subsystems Technology (RAST) server (7).

The percentage of G+C content in all contigs was 27.9%. A total of 58% of open reading frames (ORFs) (1,041) were annotatable with known proteins. The genome contained 1,786 protein-coding genes, 39 tRNA genes, and one copy of the large-subunit rRNA.

The presence of the methyl coenzyme reductase I (*mcrI*) system in JH1 likely indicates that it can grow on interspecies hydrogen transfer (8). Strain JH1 harbors genes that encode the enzymes used in energy metabolism, mainly within the methanogenesis pathway. It can grow with H_2_ plus CO_2_ and formate (*fdhA*, *fdhB*, *fdhC*) (2). Since these enzymes are present in cytoplasm, they can be used as a chemogenomic target to develop inhibitors. JH1 contains genes for exopolysaccharide production, protein glycosylation, and several adhesion-like proteins. The gene for sortase, a membrane-associated transpeptidase (*srtA*), was also identified in JH1, and its product can be used against methanogens (9). Mtr enzyme complex (MtrEDC; transfer methyl group from coenzyme M methyltransferase to coenzyme M, coupled to efflux of Na^+^ ions) was mentioned previously as a good antibody binding site (2). A similar enzyme complex was observed in the genome of JH1. Overall, the draft genome sequence of JH1 provides a better understanding of the cellular processes of genus *Methanobrevibacter*. It also provides clues regarding the functional roles of the proteins that can be targeted for the broad inhibition of rumen methanogens.

### Nucleotide sequence accession numbers.

The draft genome sequence of *Methanobrevibacter* strain JH1 is available in DDBJ/EMBL/GenBank under the accession no. BAGX02000001 to BAGX02000054.
